# Supplementary material for: Pairing interacting protein sequences using masked language modeling
Source: Proc Natl Acad Sci U S A. 2024 Jun 24;121(27):e2311887121. doi: 10.1073/pnas.2311887121 (PMC11228504; doi:10.1073/pnas.2311887121)
Supplement: Supplementary file 1 — Appendix 01 (PDF) [file pnas.2311887121.sapp.pdf]

Supplementary material for “Pairing interacting protein sequences using masked language modeling”

Umberto Lupo[1,2,*,†], Damiano Sgarbossa[1,2,*], Anne-Florence Bitbol[1,2,†]

1 Institute of Bioengineering, School of Life Sciences, École Polytechnique Fédérale de Lausanne (EPFL), CH-1015 Lausanne, Switzerland

2 SIB Swiss Institute of Bioinformatics, CH-1015 Lausanne, Switzerland

* These authors contributed equally to this work.

† Emails: umberto.lupo@epfl.ch, anne-florence.bitbol@epfl.ch

S1 Supplementary methods

S1.1 MSA Transformer and masked language modeling for MSAs

We use the MSA Transformer model [1], which takes MSAs as inputs and was trained with a variant of the masked language modeling (MLM) objective [2] on a training set of 26 million MSAs constructed from UniRef50 clusters. The model's training objective was to correctly predict the identity of randomly masked residue positions in the MSAs in its training set. Specifically, it was trained to minimize an MLM loss, which reads, for an MSA $\mathcal{M}$, and its masked version $\widetilde{\mathcal{M}}$:

$$\mathcal{L}_{\mathrm{MLM}}(\mathcal{M}, \widetilde{\mathcal{M}}; \theta) = -\sum_{(m,i)\,\in\,\mathrm{mask}} \log p(x_{m,i} \,|\, \widetilde{\mathcal{M}}; \theta)\,. \tag{S1}$$

Here, $x_{m,i}$ denotes the amino acid at the i-th residue position (column) in the m-th sequence (row) of $\mathcal{M}$, while θ stands for all the model parameters. At each residue position in the input MSA, MSA Transformer outputs a probability for each of the 21 possible amino-acid and gap symbols, and $p(x_{m,i} \,|\, \widetilde{\mathcal{M}}; \theta)$ in Eq. (S1) is the probability associated with the correct residue $x_{m,i}$ at MSA position (m, i). MSA Transformer's architecture interleaves multi-headed (tied) row attention blocks and (untied) column attention blocks, over several layers. Therefore, the accessible context for a masked residue consists not only of amino acids at different positions along the same sequence, but also of amino acids from other sequences [1, 3]. This allows the model to capture coevolution information. After pre-training, each term in the right-hand side of Eq. (S1) can be interpreted as the model's estimate of the (negative) log-likelihood of the amino acid $x_{m,i}$ at a masked position (m, i) [4–6].

S1.2 A differentiable formulation of paralog matching

We formulate a differentiable optimization problem that can be more efficiently solved than the brute-force search, using gradient methods. The goal is to obtain sets of within-species matchings (and thus permutations) that minimize our MLM loss.

The set $\mathcal{P}_N$ of permutation matrices of N objects can be parameterized exactly by square matrices X via the *matching operator*

$$M(X) = \arg\max_{P\in\mathcal{P}_N} [\mathrm{trace}(P^{\mathrm{T}} X)], \tag{S2}$$

which can be computed using standard non-differentiable algorithms for linear assignment problems [7].[1]

We exploit the fact, shown in [8], that permutation matrices can be approximated arbitrarily well by using the *Sinkhorn operator* S, which is defined on square matrices X as follows:

$$S(X) = \lim_{l\to\infty} S^l(X), \quad \text{where} \quad S^l(X) = (\mathcal{T}_\mathrm{c} \circ \mathcal{T}_\mathrm{r})^l(\exp(X)), \tag{S3}$$

$\mathcal{T}_\mathrm{c}$ and $\mathcal{T}_\mathrm{r}$ are the row- and column-wise normalization operators, and exp denotes the component-wise matrix exponential.[2] More precisely, $M(X) = \lim_{\tau\to 0^+} S(X/\tau)$ for almost all X [8, Theorem 1]. Hence, by choosing a suitably small value of τ, and using S^l [Eq. (S3)] instead of S for a suitably large l, we can define a smooth mapping $\hat{S}(X) = S^l(X/\tau)$ which sends arbitrary square matrices to "soft permutations" approximating *bona fide* ("hard") permutations. In practice, we use $\tau = 1$ and $l = 10$.

Applying general soft permutations directly on an MSA (after one-hot encoding its residues) yields a dataset consisting of "amino acid mixtures" at each MSA position. Such datasets are out of distribution relative to MSA Transformer's pre-training since it was trained on single amino acid embeddings, not mixtures of them. Besides, we wish to optimize for an MLM loss defined on realistic MSAs. Therefore, in order to be able to backpropagate through $\hat{S}$, while also evaluating MLM losses only on MSAs shuffled by hard permutations, we compute the full matching operator M [Eq. (S2)] in the forward pass, but propagate gradients backwards through $\hat{S}$ alone.[3]

S1.3 Datasets

Benchmark prokaryotic datasets. We developed and tested DiffPALM using joint MSAs extracted from two datasets. The first dataset is composed of 23,632 cognate pairs of histidine kinases (HK) and response regulators (RR) from the P2CS database [10, 11], paired using genome proximity, and previously described in [12, 13]. The average number of pairs per species in this dataset is 10.23 (standard deviation: 7.85).

The second dataset consists of 17,950 ABC transporter protein pairs, homologous to the *Escherichia coli* MALG-MALK pair of maltose and maltodextrin transporters, also paired using genome proximity [12, 14]. The average number of pairs per species in this dataset is 5.68 (standard deviation: 5.60). We also considered a similarly constructed dataset of 220 pairs homologous to the *Escherichia coli* NUOA-NUOJ pair of NADH-quinone oxidoreductase subunits, with an average number of pairs per species of 2.04.

Throughout, our focus is on pairing interaction partners among paralogs within each species. In all these benchmark datasets, species comprising only one pair of sequences were discarded. Indeed, pairing is trivial in these cases.

Out of each of these benchmark datasets of known interacting pairs, we consider paired MSAs of depth ∼50 (resp. ∼100 or ∼200), constructed by selecting all the sequences of randomly sampled species from the full dataset. Specifically, for a target MSA depth $\overline{D} = 50$, 100 or 200, we add randomly sampled complete species one by one; if the first m species (but no fewer) give an MSA depth $D \geq 0.9\overline{D}$, and the first $n \geq m$ species (but no more) give $D \leq 1.1\overline{D}$, then we select the first k species in our final MSA, with k picked uniformly at random between m

[1] More precisely, the right-hand side of Eq. (S2) has a unique solution for almost all X [8].

[2] That is, S^l consists of applying exp and then iteratively normalizing rows and columns l times.

[3] See [9] for a similar use of "gradient bypassing" in the context of protein design. We write the hard permutation as $[M(X)-\hat{S}(X)]+\hat{S}(X)$, and halt gradient backpropagation through the term in square brackets. Schematically, let L denote the operator which takes an MSA as input, randomly masks it, passes the masked MSA to MSA Transformer, and finally computes the MLM loss. Then, if A is the MSA whose rows we wish to permute, we use $L'(M(X)A)\, S'(X)A$ instead of $L'(M(X)A)\, M'(X)A$ as our "gradient".

and n. For such shallow MSAs, existing coevolution-based methods do not perform well. Note also that MSA Transformer's large memory footprint constrains the depth and length of input MSAs. Concretely, in our GPU (NVIDIA RTX A6000 with 48 GB of memory) it is possible to backpropagate the gradients for an input that has up to ∼40,000 tokens, i.e. ∼200 sequences of length ∼200 amino acids.

Eukaryotic complexes. We considered targets whose structure are not in the training set of AFM with v2 weights, and where default AFM predictions are poor. Specifically, we started from those eukaryotic targets from Table A1 of [15] and from the "Benchmark 2" dataset in [16] whose PDB structures were released after the training cutoff for the AFM v2 weights (April 30, 2018). Among those, we focused on multimers with no more than 2 different types of monomers, where both monomers come from the same species, and with paired sequences not longer than 500 amino acids, due to GPU memory constraints. Finally, we further restricted to the 15 targets with default AFM predictions yielding the poorest reported DockQ score. They are listed in Table S1. All of them are heterodimers, except 6ABO which is a heterotetramer complex made of two IFFO1 and two XRCC4 molecules. Note that we also show results for AFM with v3 weights (latest release, training cutoff September 30, 2021) in Fig. S12.

S1.4 General points on AFM

For all structure prediction tasks, we use the five pre-trained AFM models with v2 weights [16], except in Fig. S12 where v3 weights are used. We use full genomic databases and code from release v2.3.1 of the official implementation in https://github.com/deepmind/alphafold. We use no structural templates, and perform 3 recycles for each structure, without early stopping. We relax all models using AMBER.

When using all pairing methods (default AFM, DiffPALM, orthology-based), we also retained the block MSAs retrieved by the default AFM pipeline [17].

The AFM confidence score is defined as $0.8 \cdot \text{iptm} + 0.2 \cdot \text{ptm}$, where iptm is the predicted TM-score in the interface, and ptm the predicted TM-score of the entire complex [16].

S2 Supplementary tables

PDB ID	L_A	L_B	D	F_{paired}	$\langle d_p \rangle$	MSR	F_{same}	F_{pred}	D_{DiffPALM}	D_{eff}^A	D_{eff}^B
6QU1	322	48	9267	0.02	3.4	5	0.37	0.45	76	20	11
6POG	114	249	18390	0.20	30.1	3	0.03	0.22	821	114	93
6THL	240	185	3515	0.03	2.2	5	0.40	0.46	53	28	26
6L5K	98	113	15857	0.29	22.9	3	0.05	0.75	3478	402	644
6A6I	98	76	4793	0.11	3.1	5	0.30	0.48	259	57	87
5Z5K	380	66	6349	0.03	12	10	0	0.02	3	3	3
6FYH	124	76	15285	0.51	7.1	3	0.14	0.72	5550	1703	1640
6WCW	184	254	20435	0.21	6.7	3	0.18	0.57	2509	263	402
5XLN	190	45	9434	0.04	3.4	5	0.30	0.65	228	30	31
7BQU	114	28	5915	0.04	2.9	5	0.44	0.65	152	67	61
6ABO	227	82	5058	0.10	4.5	3	0.32	0.56	310	65	25
6INE	267	177	19227	0.18	4	3	0.26	0.39	1334	402	480
6IRE	234	194	7599	0.12	4.2	3	0.21	0.63	591	120	210
6PNQ	202	168	5694	0.20	8.7	5	0.13	0.25	282	117	23
6GK2	106	92	3062	0.08	2.4	5	0.35	0.59	145	20	40

Table S1: **Dataset of eukaryotic complexes.** All 15 eukaryotic protein complexes considered here are listed by their PDB ID, and various MSA properties are given. L_A and L_B are the lengths of the aligned amino acid sequences of the two chains A and B considered. D denotes the depth of the full MSA built by AFM, consisting of the paired MSA and the block MSAs (see "Assessing the impact of pairing on AFM structure prediction"). F_{paired} is the fraction of sequences that are paired by AFM, i.e. the depth of the paired MSA divided by D. $\langle d_p \rangle$ is the average depth of the MSAs of the single species in the AFM-paired MSA. Thus it is the average of the largest depth among the two chains, since the other one is completed by padding sequences of gaps. MSR stands for "maximum size ratio": if the ratio of the larger to the smaller of the depths of MSAs A and B is larger than MSR, species are not paired by DiffPALM. F_{same} is the fraction of pairs predicted by DiffPALM that is identical to the pairs predicted by the default AFM pairing method. F_{pred} is the ratio of the number of pairs predicted with DiffPALM to the number predicted using the default AFM pairing method. $D_{\text{DiffPALM}} = D \times F_{\text{paired}} \times F_{\text{pred}}$ denotes the number of pairs output by DiffPALM. D_{eff}^A (resp. D_{eff}^B) is the effective depth corrected with phylogenetic weights (with Hamming distance threshold 0.2) [18] of the MSA associated to chain A (resp. chain B) in the MSAs which are paired by DiffPALM. These effective depths quantify MSA diversity. Rows are ordered by increasing mean DockQ score for the default AFM pairing method (cf. Fig. S5).

MSAs	Pairing method	Pos. Ex.	N_{runs}	Precision-100	Precision-10
HK-RR	Chance	-	-	0.09	-
HK-RR	DCA-IPA [12]	0	-	0.16	-
HK-RR	MI-IPA [13]	0	-	0.15	-
HK-RR	GA-IPA [19]	0	-	0.18	-
HK-RR	ESM-2 (650M)	0	-	0.11 – 0.16	-
HK-RR	DiffPALM-MRA	0	5	0.37	0.67
HK-RR	DiffPALM-MRA	0	20	0.39	0.71
HK-RR	DiffPALM-IPA	0	20 + 10	0.44	-
HK-RR	DiffPALM-MRA	11	5	0.51	0.87
HK-RR	DiffPALM-MRA	19	5	0.61	0.95
HK-RR	DiffPALM-MRA	45	5	0.74	0.99
MALG-MALK	Chance	-	-	0.20	-
MALG-MALK	DCA-IPA [12]	0	-	0.31	-
MALG-MALK	MI-IPA [13]	0	-	0.32	-
MALG-MALK	GA-IPA [19]	0	-	0.42	-
MALG-MALK	ESM-2 (650M)	0	-	0.19 – 0.31	-
MALG-MALK	DiffPALM-MRA	0	5	0.55	0.84

Table S2: **Performance of pairing by DiffPALM vs. baselines.** We report the pairing precision for variants of DiffPALM, namely MRA & IPA with various numbers of positive examples (Pos. Ex.) and runs (N_{runs}), as well as for different baseline methods, on 40 MSAs comprising about 50 HK-RR or MALG-MALK pairs. With all methods, a full one-to-one within-species pairing is produced for each MSA, and performance is measured by precision, namely, the fraction of correct pairs among predicted pairs, averaged over the 40 MSAs considered. In "precision-100", this fraction is computed over all predicted pairs (100% of them). In "precision-10", it is calculated over the top 10% predicted pairs, when ranked by predicted confidence. For the IPA method, we use 20 runs of MRA as starting point, and we add 5 fixed pairs at each new run, see "DiffPALM: Paralog matching based on MLM". The chance expectation, and the performance of DCA-IPA [12], MI-IPA [13], and GA-IPA [19] are reported as baselines. We also consider a pairing method based on the scores given by the ESM-2 (650M) protein language model [20], see "Pairing based on a single-sequence language model". For this method, we consider 10 different values of masking probability from 0.1 to 1.0, and we report the range of precisions obtained.

S3 Supplementary figures

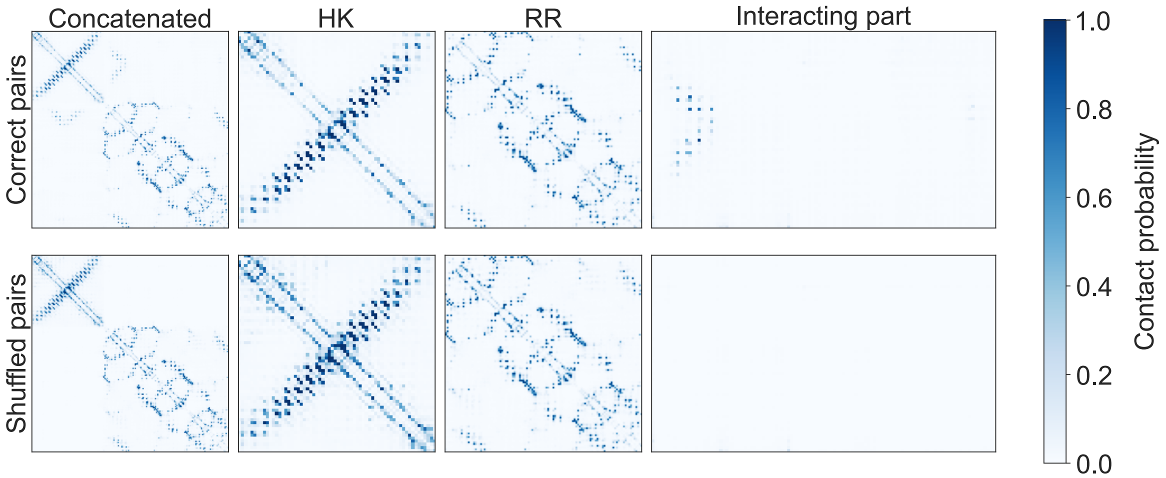

Figure S1: **Comparison of contact maps predicted by MSA Transformer for the correct pairing of an HK MSA and an RR MSA ("Correct pairs"), and for an incorrect pairing ("Shuffled pairs").** We observe that MSA Transformer is able to correctly predict the inter-protein contacts when given as input a paired MSA made of correctly matched sequences. Conversely, if the model is given as input a paired MSAs where rows have been shuffled before pairing, it is not able to recover the inter-protein contact map (even though it correctly recovers correctly the intra-protein contact maps). These results suggests that MSA Transformer can distinguish between interacting and non-interacting pairs of protein sequences, despite the fact that dimers or paired MSAs were not in the training set used for its MLM pre-training [1].

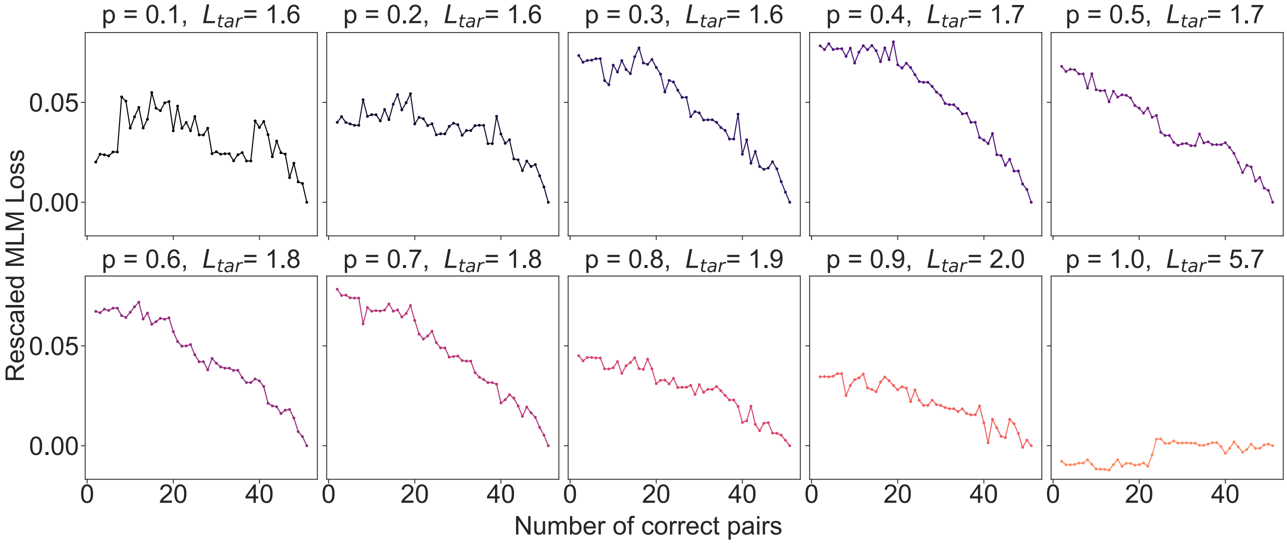

Figure S2: **MLM loss vs. number of correct pairs for different masking probabilities.** We use an MSA of 50 correctly paired sequences comprising 5 different species from the HK-RR dataset. To estimate the expected loss accurately, we used 20 different masks at each step. L_{tar} denotes the expected loss when all pairs are correctly matched. For visualization purposes, in every plot we rescale the loss by shifting it by L_{tar}. We find that our MLM loss in Eq. (S1) decreases for increasing numbers of correctly matched sequences in the MSA. We see that the sweet spot of the masking probability p (i.e. the value that gives steeper and smoother loss curves) is at moderately high values ($0.4 \leq p \leq 0.8$). As explained in "Methods", high masking probabilities make it more challenging for the model to predict the masked amino acids using only information coming from the masked MSA, thus encouraging it to use, instead, information coming from the matched MSA. This motivates our choice of a masking probability of $p \geq 0.7$.

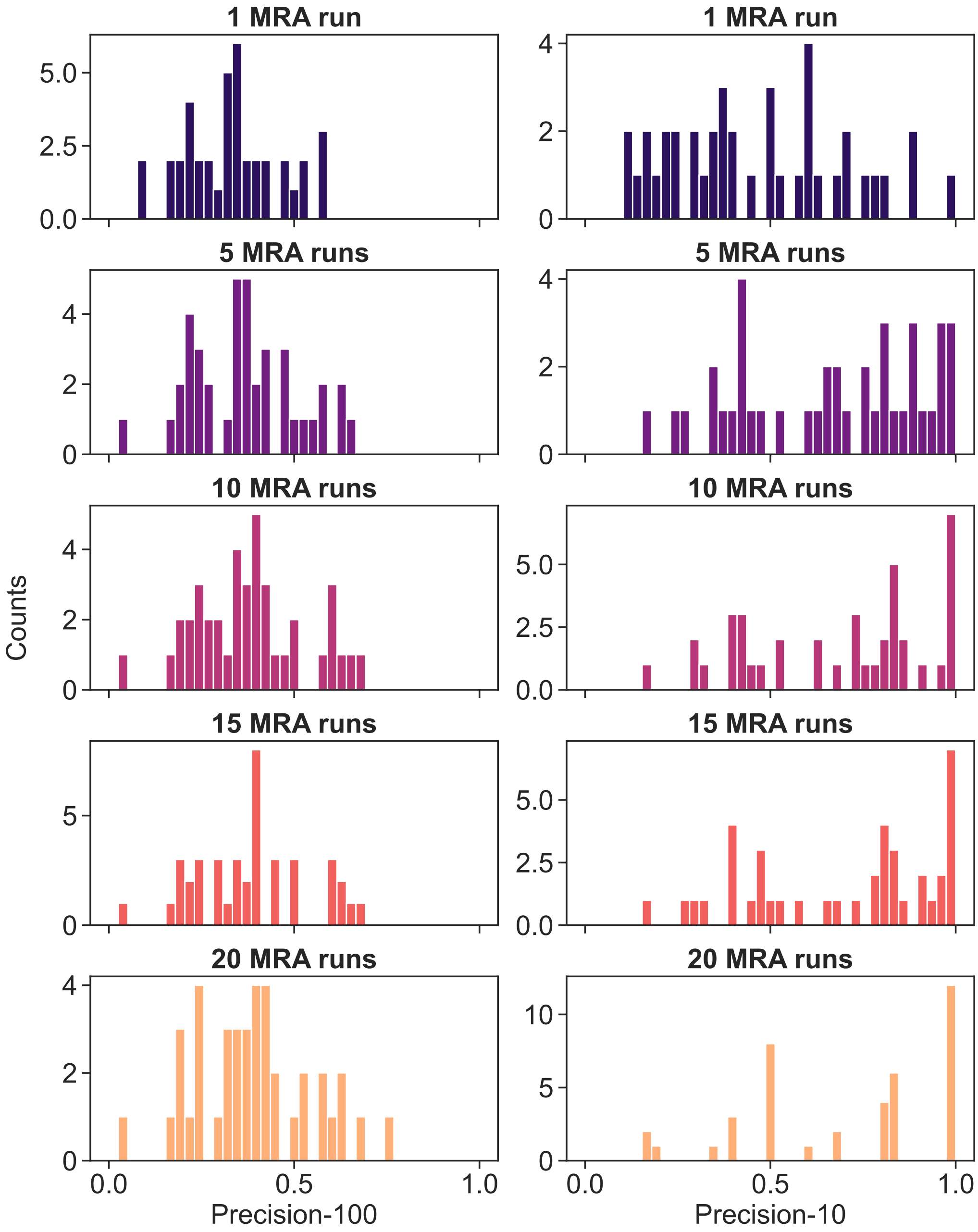

Figure S3: **Distribution of precision scores for different number of MRA runs.** We report the histograms of precision scores obtained by the MRA variant of DiffPALM on each of the 40 MSAs comprising about 50 HK-RR pairs (used in "DiffPALM outperforms other coevolution methods on small MSAs"), for different number of MRA runs. Precision-100 and precision-10 are defined in Fig. 1 and Table S2. We observe a skewed distribution for precision-10 scores, especially after many MRA runs: a very high precision is reached for many MSAs, but low precisions are obtained for some. Fig. 1 displays the average and the standard error of each of the distributions shown here.

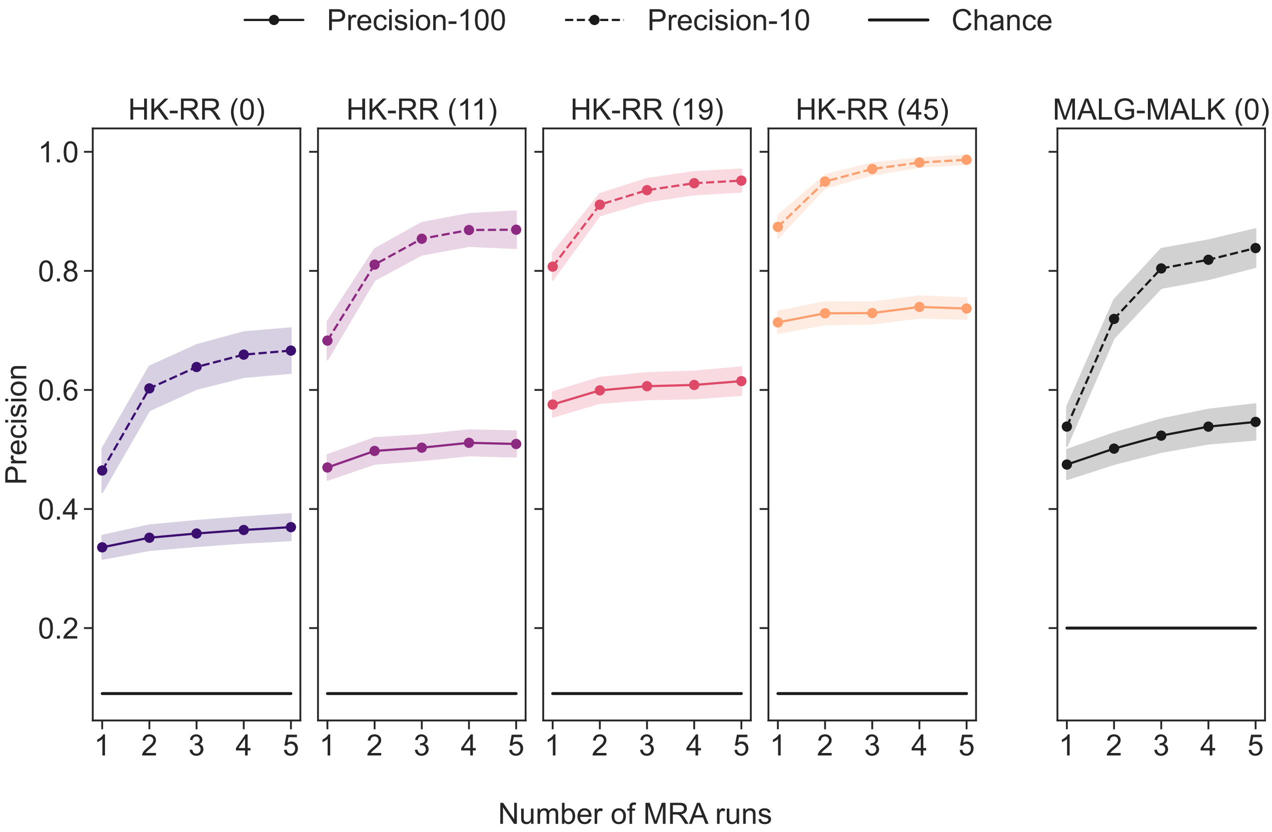

Figure S4: **Performance of DiffPALM for different numbers of positive examples and for two pairs of protein families.** The performance of DiffPALM is plotted versus the number of MRA runs. As in Fig. 2, we compare runs with various numbers of positive examples (left panels) on the 40 MSAs comprising about 50 HK-RR pairs (used in "DiffPALM outperforms other coevolution methods on small MSAs"), and runs on 40 MSAs comprising about 50 MALG-MALK pairs with no positive example (right panel). Precision-100 and precision-10 are defined in Fig. 1 and Table S2. The protein families considered and the number of positive examples are indicated in the title of each panel (the latter between brackets). In each case, we plot the mean value over the 40 different MSAs considered and the standard error interval. The chance expectation is shown for reference.

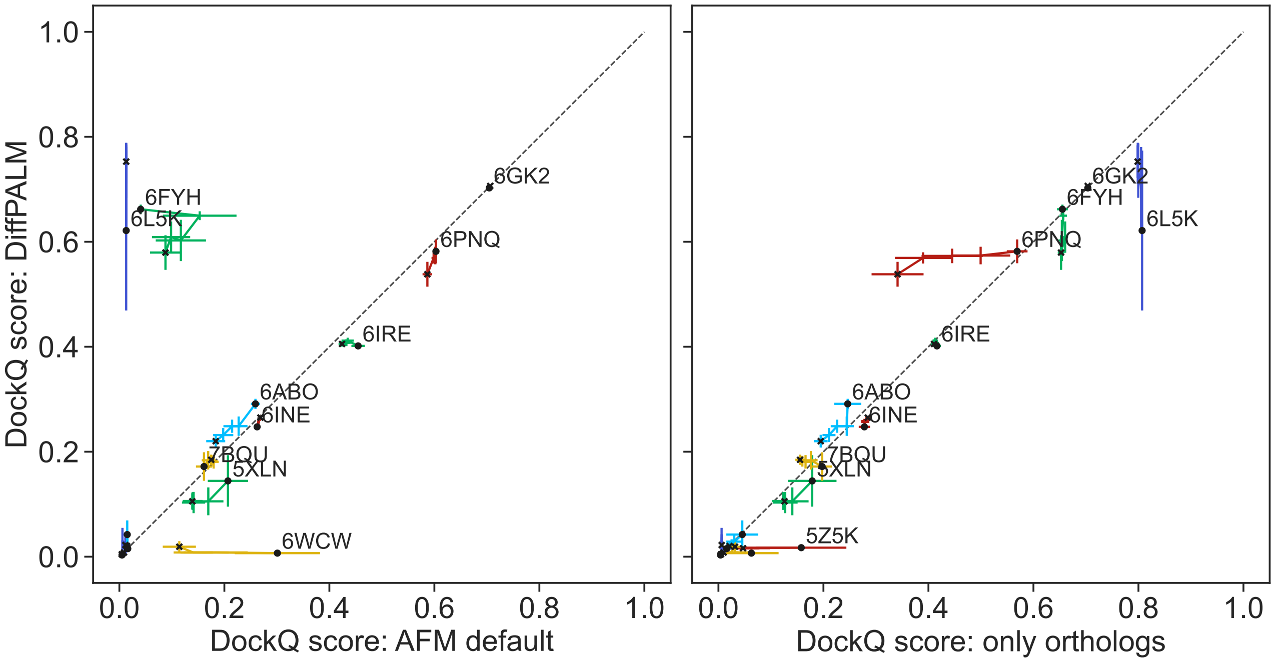

Figure S5: **Performance of structure prediction by AFM using different MSA pairing methods.** We report the performance of AFM, in terms of DockQ scores, for the 15 complexes listed in Table S1, using three different pairing methods on the same initial unpaired MSAs. Left panel: DiffPALM versus default AFM pairing. Right panel: DiffPALM versus only pairing orthologs to the two query sequences. As in Fig. 3, for each complex, AFM is run five times, and the five top predicted structures by AFM confidence are considered each time, yielding 25 predicted structures total. For each complex, we show "trajectories" of performance starting from the top-confidence predicted structure (black circular marker) and ending with all predicted structures up to and including the fifth one (black cross marker). Results are averaged over the 5 runs and standard errors are shown as error bars. Points with DockQ below 0.1 are not labelled with their PDB ID for graphical reasons. Note that Fig. 3 restricts to those complexes where any two of these three pairing methods yield a significant difference (> 10%) in average DockQ scores, among those shown here and listed in Table S1.

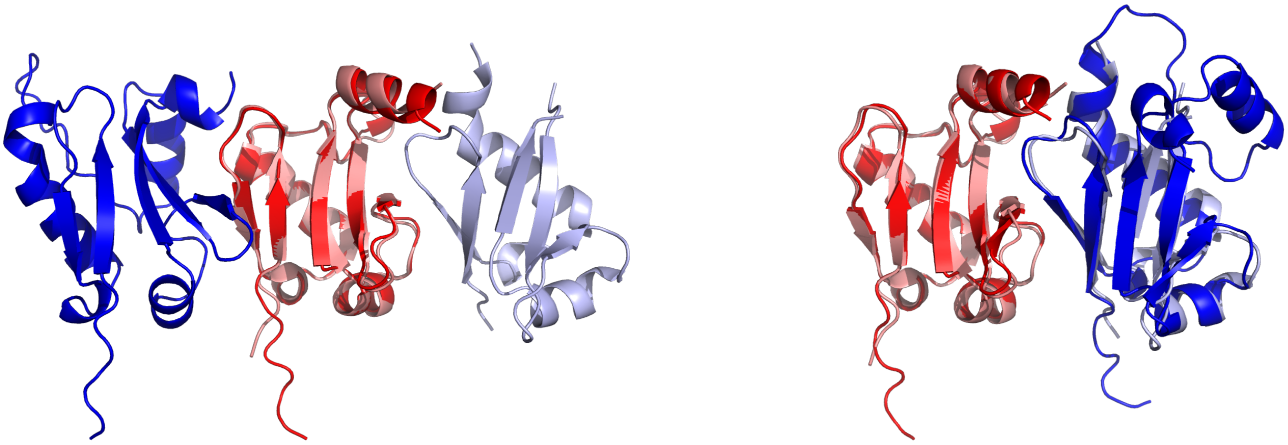

(a) AFM default: DockQ = 0.013.

(b) DiffPALM: DockQ = 0.811.

Figure S6: **Comparing the AFM default MSA pairing strategy with DiffPALM, for structure 6L5K.** In both panels, we superimpose the experimental structure of 6L5K with a structure predicted using AFM. Chains A and B of the PDB structure are colored in salmon and light blue respectively, while chains A and B of both predicted structures are colored in bright red and bright blue respectively. (a) Comparing the experimental structure with a typical high-confidence prediction generated with the default MSA pairing pipeline. (b) Comparing the experimental structure with a typical high-confidence prediction generated with our MSA pairing pipeline based on DiffPALM.

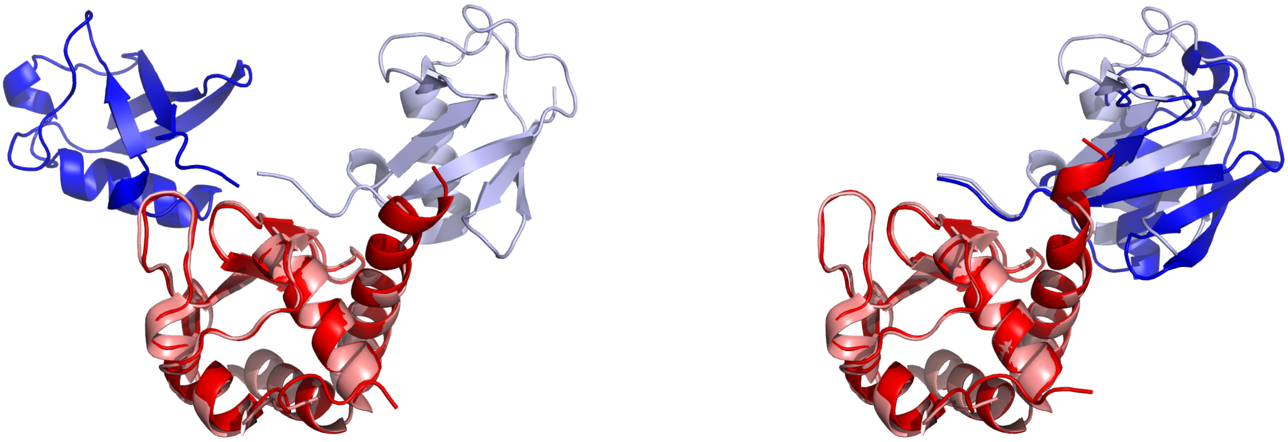

(a) AFM default: DockQ = 0.044.

(b) DiffPALM: DockQ = 0.669.

Figure S7: **Comparing the AFM default MSA pairing strategy with DiffPALM, for structure 6FYH.** Same as Fig. S6, but for 6FYH.

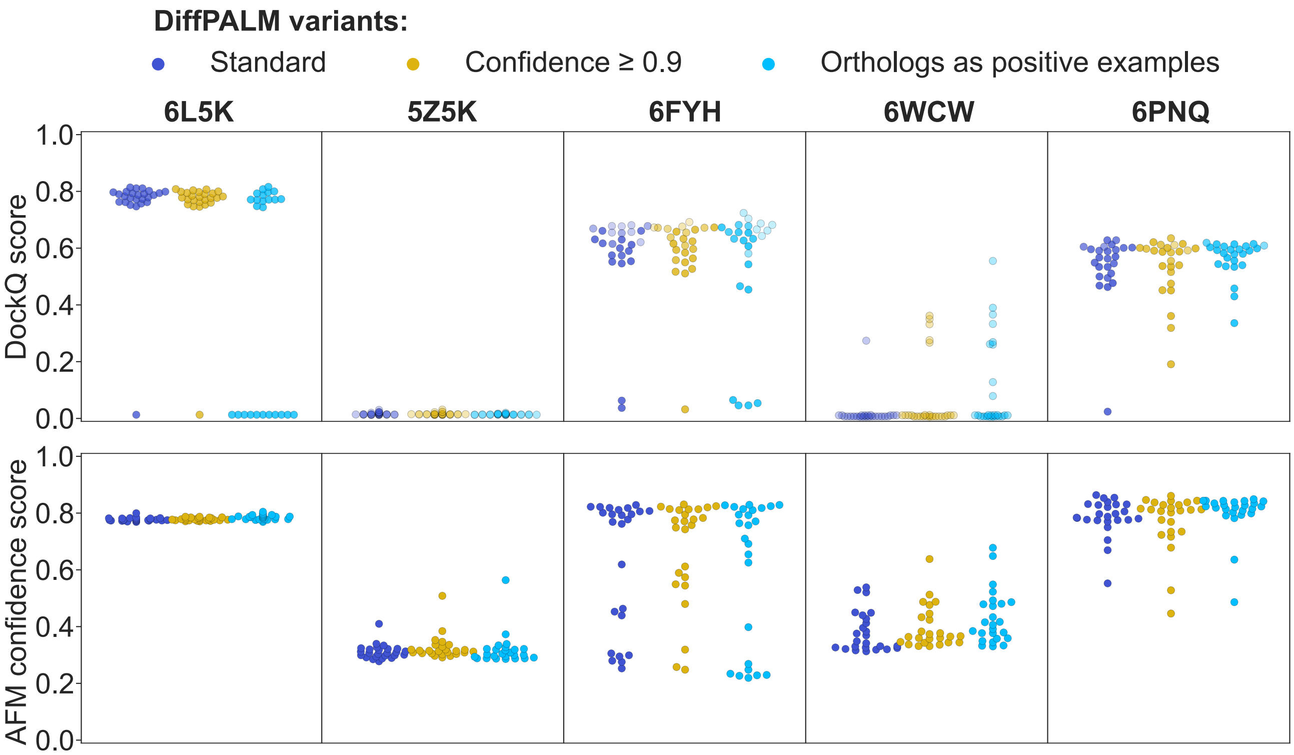

Figure S8: **Performance of AFM using different variants of DiffPALM.** Same as Fig. 3, but here we compare the standard DiffPALM method with two of its variants: one where we only use pairs with high predicted confidence (≥ 0.9) as input to the AFM pipeline, and one where we use orthology-based pairs (i.e. those employed in the "Only orthologs" case shown in Fig. 3) as positive examples for DiffPALM, and use the pairs predicted by DiffPALM, as well as the positive examples, as input of AFM.

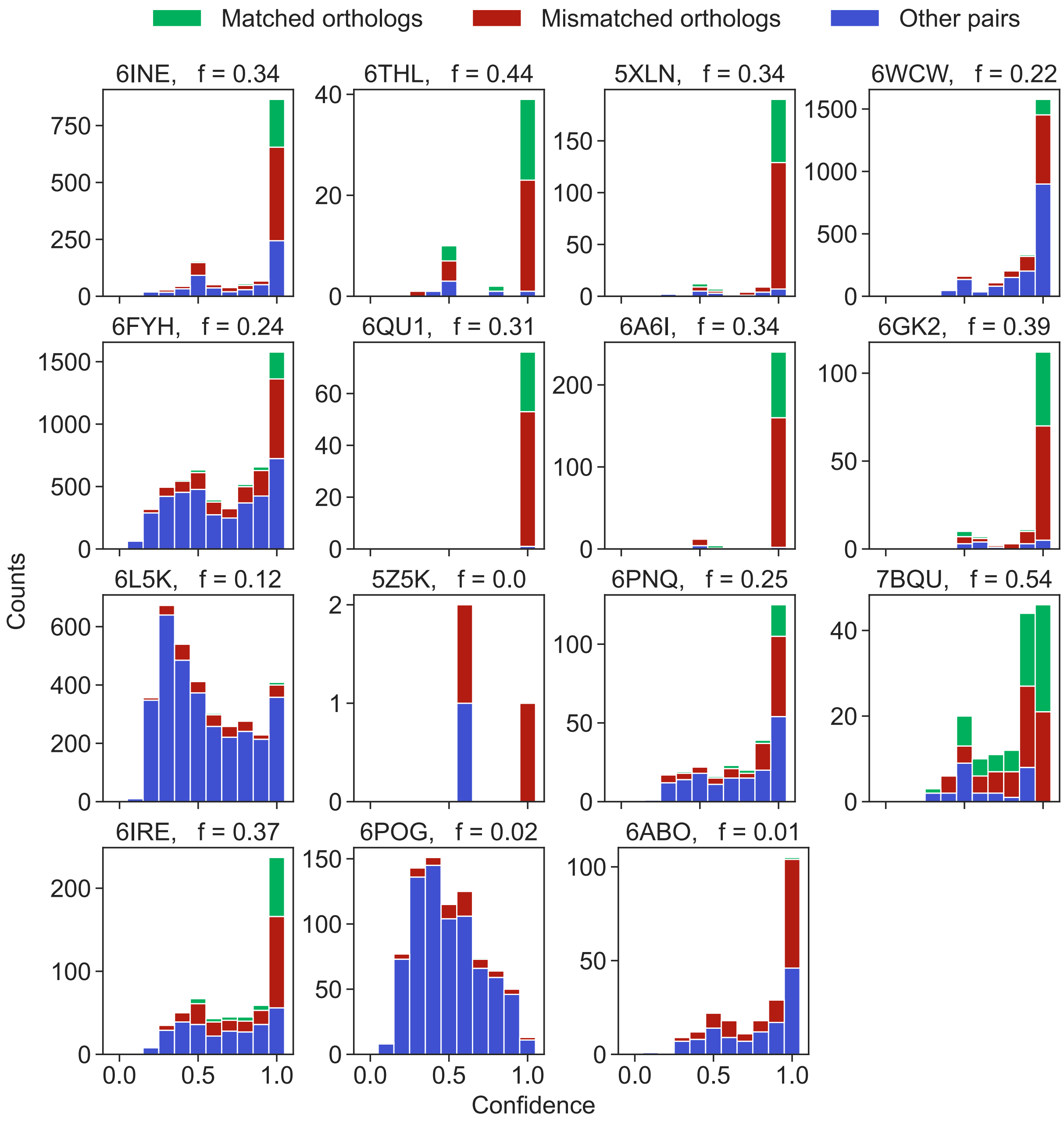

Figure S9: **Confidence of DiffPALM predictions.** We show, for the 15 complexes listed in Table S1, histograms of the DiffPALM confidence values (see "Result and confidence"). We distinguish the orthology-based pairs that are recovered by DiffPALM, the otherwise paired orthologs, and all the other paired sequences. We indicate in panel titles the value of the fraction f of orthology-based pairs that are recovered by DiffPALM.

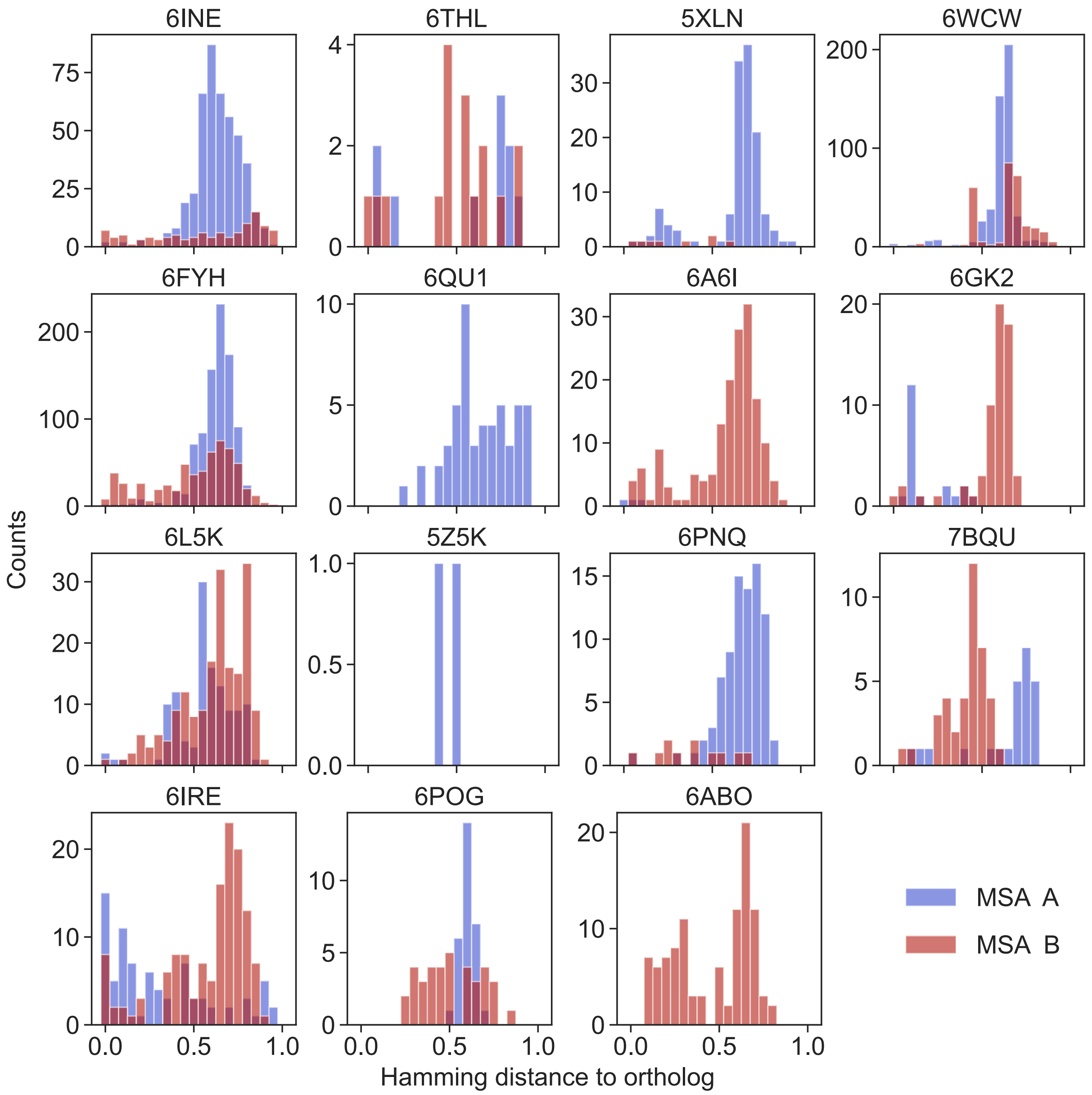

Figure S10: **Hamming distance to the orthologs of all the mismatched pairs predicted by DiffPALM.** We show, for the 15 complexes listed in Table S1, histograms of the Hamming distance between the partner predicted by DiffPALM and the one predicted by matching orthologs to the query sequences, whenever they differ. In practice, for each sequence A_1 in family A which is paired with a partner B_1 from family B using orthology, but with a different partner B_2 using DiffPALM, we measure the Hamming distance between B_1 and B_2. A similar protocol is conducted for each sequence B_1 in family B. These distances allow us to compare the pairs predicted by DiffPALM to the orthology-based pairs. Note that the total counts of the distributions regarding MSA A and MSA B generally differ. This happens because DiffPALM might pair orthologs to padding sequences of gaps: in this case, we do not report Hamming distances.

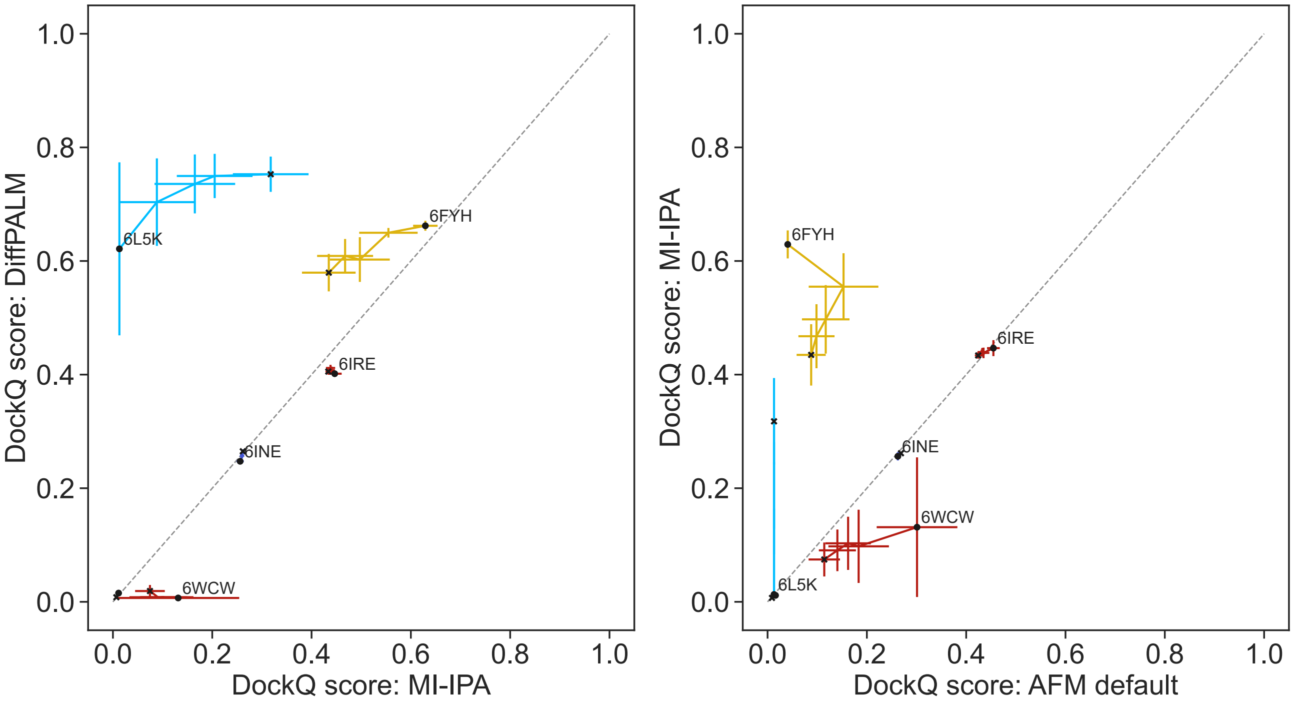

Figure S11: **Performance of structure prediction by AFM using MI-IPA pairing.** Same as Fig. S5 but comparing DiffPALM to MI-IPA (left panel) and MI-IPA to the default AFM pairing method (right panel). Here we restricted to the 6 eukaryotic complexes with deepest pairable MSAs (see Table S1), due to the depth requirements of traditional coevolution methods.

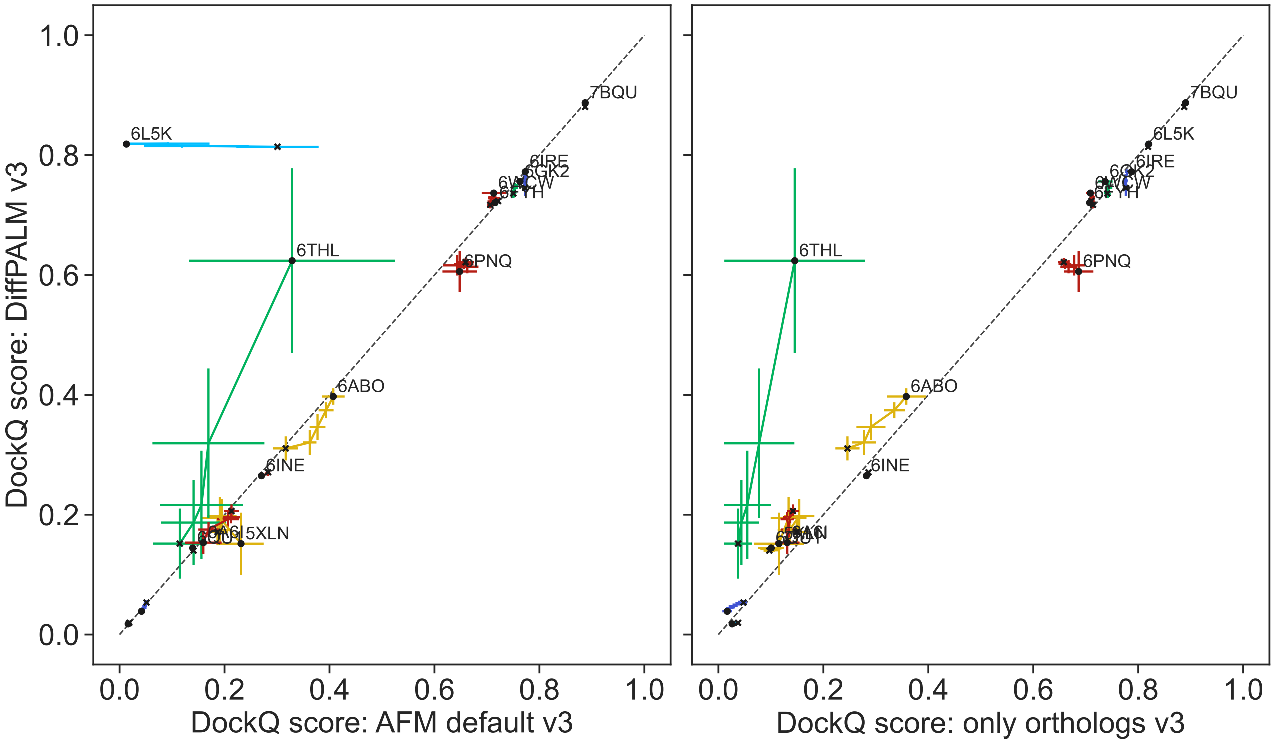

Figure S12: **Performance of structure prediction by AFM-v3 using different MSA pairing methods.** Same as Fig. S5 but using AlphaFold-Multimer with v3 weights (recall that v2 weights are used in the rest of this work).

References

[1] R. M. Rao, J. Liu, R. Verkuil, J. Meier, J. Canny, P. Abbeel, T. Sercu, and A. Rives, "MSA Transformer," in *Proceedings of the 38th International Conference on Machine Learning*, vol. 139. PMLR, 2021, pp. 8844–8856. [Online]. Available: https://proceedings.mlr.press/v139/rao21a.html

[2] J. Devlin, M.-W. Chang, K. Lee, and K. Toutanova, "BERT: Pre-training of deep bidirectional transformers for language understanding," in *Proceedings of the 2019 Conference of the North American Chapter of the Association for Computational Linguistics: Human Language Technologies, Volume 1 (Long and Short Papers)*. Minneapolis, Minnesota: Association for Computational Linguistics, 2019, pp. 4171–4186.

[3] U. Lupo, D. Sgarbossa, and A.-F. Bitbol, "Protein language models trained on multiple sequence alignments learn phylogenetic relationships," *Nat Commun*, vol. 13, no. 6298, 2022.

[4] A. Wang and K. Cho, "BERT has a mouth, and it must speak: BERT as a Markov random field language model," *arXiv*, 2019.

[5] K. Goyal, C. Dyer, and T. Berg-Kirkpatrick, "Exposing the implicit energy networks behind masked language models via Metropolis–Hastings," *arXiv*, 2021.

[6] R. Rao, J. Meier, T. Sercu, S. Ovchinnikov, and A. Rives, "Transformer protein language models are unsupervised structure learners," in *International Conference on Learning Representations*, 2021. [Online]. Available: https://openreview.net/forum?id=fylclEqgvgd

[7] H. W. Kuhn, "The Hungarian method for the assignment problem," *Naval Research Logistics Quarterly*, vol. 2, pp. 83–97, 1955.

[8] G. E. Mena, D. Belanger, S. Linderman, and J. Snoek, "Learning latent permutations with Gumbel-Sinkhorn networks," *6th International Conference on Learning Representations, ICLR 2018 - Conference Track Proceedings*, pp. 1–22, 2018. [Online]. Available: https://openreview.net/forum?id=Byt3oJ-0W

[9] C. Norn, B. I. M. Wicky, D. Juergens, S. Liu, D. Kim, D. Tischer, B. Koepnick, I. Anishchenko, F. Players, D. Baker, and S. Ovchinnikov, "Protein sequence design by conformational landscape optimization," *Proc. Natl. Acad. Sci. U.S.A.*, vol. 118, no. 11, p. e2017228118, 2021.

[10] M. Barakat, P. Ortet, C. Jourlin-Castelli, M. Ansaldi, V. Mejean, and D. E. Whitworth, "P2CS: a two-component system resource for prokaryotic signal transduction research," *BMC Genomics*, vol. 10, p. 315, 2009.

[11] M. Barakat, P. Ortet, and D. E. Whitworth, "P2CS: a database of prokaryotic two-component systems," *Nucleic Acids Research*, vol. 39, no. Database issue, pp. D771–776, 2011.

[12] A.-F. Bitbol, R. S. Dwyer, L. J. Colwell, and N. S. Wingreen, "Inferring interaction partners from protein sequences," *Proc. Natl. Acad. Sci. U.S.A.*, vol. 113, no. 43, pp. 12 180–12 185, 2016.

[13] A.-F. Bitbol, "Inferring interaction partners from protein sequences using mutual information," *PLoS Comput. Biol.*, vol. 14, no. 11, p. e1006401, 2018.

[14] S. Ovchinnikov, H. Kamisetty, and D. Baker, "Robust and accurate prediction of residue-residue interactions across protein interfaces using evolutionary information," *eLife*, vol. 3, p. e02030, 2014.

[15] B. Chen, Z. Xie, J. Qiu, Z. Ye, J. Xu, and J. Tang, "Improved the heterodimer protein complex prediction with protein language models," *Briefings in Bioinformatics*, vol. 24, no. 4, p. bbad221, 2023.

[16] R. Evans, M. O'Neill, A. Pritzel, N. Antropova, A. Senior, T. Green, A. Žídek, R. Bates, S. Blackwell, J. Yim, O. Ronneberger, S. Bodenstein, M. Zielinski, A. Bridgland, A. Potapenko, A. Cowie, K. Tunyasuvunakool, R. Jain, E. Clancy, P. Kohli, J. Jumper, and D. Hassabis, "Protein complex prediction with AlphaFold-Multimer," *bioRxiv*, 2021.

[17] P. Bryant, G. Pozzati, and A. Elofsson, "Improved prediction of protein-protein interactions using AlphaFold2," *Nat Commun*, vol. 13, no. 1, p. 1265, 2022.

[18] M. Weigt, R. A. White, H. Szurmant, J. A. Hoch, and T. Hwa, "Identification of direct residue contacts in protein-protein interaction by message passing," *Proc. Natl. Acad. Sci. U.S.A.*, vol. 106, no. 1, pp. 67–72, 2009.

[19] C. A. Gandarilla-Perez, S. Pinilla, A.-F. Bitbol, and M. Weigt, "Combining phylogeny and coevolution improves the inference of interaction partners among paralogous proteins," *PLoS Comput. Biol.*, vol. 19, no. 3, p. e1011010, 2023.

[20] Z. Lin, H. Akin, R. Rao, B. Hie, Z. Zhu, W. Lu, N. Smetanin, R. Verkuil, O. Kabeli, Y. Shmueli, A. dos Santos Costa, M. Fazel-Zarandi, T. Sercu, S. Candido, and A. Rives, "Evolutionary-scale prediction of atomic-level protein structure with a language model," *Science*, vol. 379, no. 6637, pp. 1123–1130, 2023.
